# Supplementary material for: The role of nodes in controlling and observing complex networks
Source: PLoS One. 2025 Jun 12;20(6):e0325824. doi: 10.1371/journal.pone.0325824 (PMC12161543; doi:10.1371/journal.pone.0325824)
Supplement: Attachment — Submitted filename: Response to Reviewers.pdf [file pone.0325824.s002.pdf]

Response for PONE-D-25-05744

Dear Editors and Reviewers,

I sincerely appreciate your comments and helpful suggestions to improve our paper. Based on the comments of reviewers, we have carefully revised our manuscript. Also, I have answered the reviewers' questions point by point. I hope the revised manuscript is satisfactory now. Once again, thank you for your help with processing our paper.

Best wishes,

The author: Longlong Wu

Responses to the First Reviewer

In this paper, authors have studied the role of nodes in controlling and observing complex networks. This paper is written concisely and the topic sounds interesting. But I still have some questions about the paper and my detailed comments are as follows.

Response: Thank you for your positive comments. We have answered the questions point by point and revised our manuscript according to your suggestions.

1. The authors have categorized the nodes into four categories. Did you invent this classification? What is the basis for such a categorization?

Response: Thank you for your helpful discussions. This work is grounded in the structural controllability theory [1] of complex networks. The concept of driver nodes was first introduced in [2]. Subsequently, the dual notion of sensor nodes emerged in studies on the observability of complex networks [3].

Through systematic simulations of both real and model networks, we demonstrate that in the networks satisfying structural controllability and observability, certain nodes simultaneously function as both driver and sensor nodes. Conversely, we also identify nodes that serve neither role. Based on these findings, we divide the nodes into four categories: (1) driver nodes, (2) sensor nodes, (3) dual-identity nodes (exhibiting both driver and sensor properties), and (4) ordinary nodes (serving neither function).

[1] Lin, C. T., Structural controllability, IEEE Transactions on Automatic Control, vol. 19, 1974, pp. 201-208.
[2] Liu, Y.-Y., Slotine, J.-J. and Barabási, A.-L., Controllability of complex networks, Nature, vol. 473, 2011, pp. 167-173.
[3] Liu, Y.-Y., Slotine, J.-J. and Barabási, A.-L., Observability of complex systems, Proceedings of the National Academy of Sciences, vol. 110, 2013, pp. 2460–2465.

2. In Introduction, I can only see that this topic has a certain degree of research ability, but it does not highlight the innovation of this paper.

Response: Thank you for your helpful suggestion. We revised the last part of the introduction to highlight the innovation of the paper. The modifications are as follows:

In this paper, we propose a novel framework for characterizing the functional roles of nodes in the structural controllability and observability of complex networks. Specifically, we propose an innovative classification scheme in which nodes are categorized based on their distinct roles in network control and observation: (1) driver nodes, which exert control the network dynamics, (2) sensor nodes, responsible for network observability, (3) dual-identity nodes, simultaneously functioning as both driver and sensor nodes, and (4) ordinary nodes, which do not participate directly in control or observation. Leveraging graph-theoretic principles, we develop a maximum matching-based framework capable of systematically identifying all four node categories in arbitrary directed networks. A key advancement of our approach is that all node types can be determined through a single maximum matching operation, significantly enhancing computational

efficiency and theoretical clarity.

Through a comprehensive analysis of various real and model networks, we have several fundamental findings. First, we demonstrate the ubiquity of all four node categories in different network topologies, with dual-identity nodes representing a previously unrecognized but crucial component. Second, we further investigate the structural characteristics of four node categories and find that nodes involved in controllability and observability tend to prefer low-degree nodes, driver nodes preferentially select divergent (high out-degree) nodes, and sensor nodes tend to select convergent (high in-degree) nodes. More significantly, we establish that the proportions of four node categories are fundamentally governed by the network's degree distribution. Building upon these empirical observations, we develop a novel theoretical framework that enables the analytical prediction of category proportions directly from a network's degree distribution. Together, the proposed classification scheme and analytical tools open up new avenues for research in network science and control theory.

3. The paper lacks relevant literature citations. For example, Equation (2) and Equation (3) are derived without citation or proof.

Response: Thank you for your helpful suggestion. We have added references to Equation (2) and Equation (3) in the revised manuscript.

4. The authors obtained theoretical results by using the maximum matching method, what is the basis for using this method?

Response: Thank you for your helpful discussions. The maximum matching method provides an effective solution to the minimum input problem in structural controllability [1]. The minimum input problem concerns the identification of both the minimal number and correct locations of driver nodes required for the network to be structural controllable. In their work, the authors transformed the minimum input problem into a directed graph matching problem and subsequently solve the minimum input problem with the maximum matching method.

Inspired by the methodology of [1], we leverage graph-theoretic principles to develop a maximum matching-based framework for systematically classifying all four node categories in arbitrary directed networks (see Lemmas 1 and 2, and Theorem 1). Furthermore, we demonstrate that the proportion of these node types is primarily governed by the network's degree distribution. Building on this finding, we derive theoretical results using core percolation theory.

[1] Liu, Y.-Y., Slotine, J.-J. and Barabási, A.-L., Controllability of complex networks, Nature, vol. 473, 2011, pp. 167-173.

5. I noticed that the authors have simulated in all 18 real networks. Can the theoretical results be visualized with real examples? Or do the theoretical results in this paper have any physical meaning?

Response: Thank you for your helpful discussions. To investigate how network topology affects node classification, we conducted simulations using 18 real networks, analyzing the proportion of driver nodes, sensor nodes, DS nodes, and ordinary nodes. This paper does not discuss the

physical meaning of the theoretical results in real networks. We note that some studies have successfully implemented structural controllability theory in real network systems [1-3]. Building on these foundations, our future work will explore applications of our theoretical framework to real network systems.

[1] Gu, S., Pasqualetti, F., Cieslak, M., Telesford, Q. K., Yu, A. B., Kahn, A. E. and Bassett, D. S., Controllability of structural brain networks. Nature communications, 6(1), 2015, pp. 8414.
[2] Yan, G., Vértes, P. E., Towlson, E. K., Chew, Y. L., Walker, D. S., Schafer, W. R., and Barabási, A. L., Network control principles predict neuron function in the Caenorhabditis elegans connectome. Nature, 550(7677), 2017, pp. 519-523.
[3] Cagua, E. F., Wootton, K. L., and Stouffer, D. B., Keystoneness, centrality, and the structural controllability of ecological networks. Journal of Ecology, 107(4), 2019, pp. 1779-1790.

Responses to the Second Reviewer

Your manuscript provides a meaningful contribution to network controllability and observability by introducing a clear node classification framework and analytical methods. The findings are well-supported by simulations, and the symmetry between driver and sensor nodes is an interesting insight. Below are suggestions to improve the manuscript.

Response: Thank you for your positive comments. We have answered the questions point by point and revised our manuscript according to your suggestions.

1. DS Node Identification. The heuristic for DS nodes (based on in-leaves/out-leaves) is plausible but lacks theoretical justification (equation 10). Provide a more rigorous derivation.

Response: Thank you for your helpful discussion. As noted, equation 10 is plausible but lacks theoretical justification. Through extensive simulations of both real and model networks, we derived the empirical equation 10 by summarizing structural characteristics of DS nodes. However, this represents an estimation rather than a rigorous derivation based on the GLR procedure and core percolation theory. At present, we are unable to give a rigorously derived theoretical formula for the proportion of DS nodes. This will be the focus of our future research. At the same time, to improve transparency, we have revised the derivation process of equation 10 to explicitly clarify its empirical nature. The modifications are as follows:

Next, through extensive simulations of real and model networks, we derive an analytical estimate of the proportion of DS nodes by observing and summarizing the structural characteristics of DS nodes. Specifically, DS nodes are both driver nodes and sensor nodes, which makes them unmatched nodes in both the left and right node sets of $H(A)$*. Let node* v *with* $k_v^- = 1$ *and* $k_v^+ = 0$ *be classified as an in-leaf, and let the node with* $k_v^- = 0$ *and* $k_v^+ = 1$ *be classified as an out-leaf. Through numerous matching simulations, we observed that when multiple in-leafs share the same upstream neighbor node, these in-leafs have a high probability of being DS nodes. Similarly, when multiple out-leafs share the same downstream neighbor node, these out-leafs have a high probability of being DS nodes. Additionally, isolated nodes must be DS nodes. Based on the above observations and summaries, the proportion of DS nodes can be roughly estimated using the following formula*

$$n_{\text{DS}} = P(0^-)P(0^+) + P(0^-)P(1^+) + P(1^-)P(0^+). \quad (1)$$

2. Scalability. The maximum matching approach (Hopcroft-Karp) has known complexity limits for very large/dense networks. Discuss how the framework performs on networks with millions of nodes or high average degree.

Response: Thank you for your helpful discussion. The Hopcroft-Karp algorithm is a well-known method for finding maximum matchings in bipartite graphs. Its time complexity is $O(\sqrt{|V|}|E|)$ *(where* $|V|$ *is the number of nodes and* $|E|$ *is the number of edges), which makes it efficient for sparse or moderately dense graphs. However, its performance can degrade significantly for networks with millions of nodes or high average degree.*

The networks analyzed in this paper, both real and modeled, are relatively small and sparse,

enabling efficient node categorization using the Hopcroft-Karp algorithm. However, for large-scale networks with millions of nodes and high average degrees, the traditional Hopcroft-Karp algorithm may encounter computational and memory constraints. Therefore, we have included a description of the Hopcroft-Karp algorithm's computational complexity and applicable scope in the revised manuscript as follows:

The maximum matching can be computed using the Hopcroft-Karp [1] algorithm, which has a time complexity of $O(\sqrt{|V|}|E|)$*, where* $|V|$ *and* $|E|$ *denote the number of nodes and edges, respectively. This algorithm is highly efficient for sparse or moderately dense digraphs, but its performance degrades as network size and edge density increase.*

[1] Hopcroft, J. E., and Karp, R. M., An $n^{5/2}$ algorithm for maximum matchings in bipartite graphs. SIAM Journal on computing, 2(4), 1973, pp. 225-231.

3. Reproducibility. While the methodology is well-explained, sharing pseudocode or code for key steps (e.g., GLR procedure, bipartite matching) would aid reproducibility. Suggestion: Include an appendix or link to an open-source implementation.

Thank you for your helpful suggestions. In this study, we employ a bipartite matching algorithm to characterize node roles in controlling and observing networks. Our theoretical framework is grounded in core percolation theory, which originates from the greedy leaf removal (GLR) algorithm. As requested, I have included links to open-source implementations of both algorithms in the revised manuscript. Additionally, all relevant data have been uploaded to the supporting information file.

4. Clarity: The writing is clear, but some technical passages (e.g., GLR procedure) could benefit from additional examples.

Response: Thank you for your helpful suggestion. We use the directed graphs and their bipartite representations in Figs. 1(a) and (b) of the original manuscript as examples to further demonstrate the GLR procedure. The revised content is as follows:

The dependence of n_{D}*,* n_{S}*,* n_{DS}*, and* n_{O} *on the degree distribution enables us to derive their analytical expressions. The theoretical framework is based on the core penetration theory, which is derived from the greedy leaf removal (GLR) procedure. In this procedure, each iteration removes: (1) a node with degree one (leaf) along with its sole nearest neighbor (root), and (2) the connecting edge, which is added to the matching. When multiple leaves connect to the same root, the algorithm arbitrarily selects one such pair for removal. For example, Figs. 1(a) and (b) give a digraph* $G(A)$ *and its bipartite representation* $H(A)$*, respectively . In* $H(A)$*, nodes* x_2^r *and* x_3^r *are leaves connected to root* x_1^l*. The GLR procedure would remove leaf* x_2^r *and root* x_1^l*, subsequently adding edge* (x_1^l, x_2^r) *to the matching.*
